# NAPPA as a Real New Method for Protein Microarray Generation

**DOI:** 10.3390/microarrays4020214

**Published:** 2015-04-24

**Authors:** Paula Díez, María González-González, Lucía Lourido, Rosa M. Dégano, Nieves Ibarrola, Juan Casado-Vela, Joshua LaBaer, Manuel Fuentes

**Affiliations:** 1 Department of Medicine and General Cytometry Service-Nucleus, Cancer Research Centre (IBMCC/CSIC/USAL/IBSAL), Salamanca 37007, Spain; E-Mails: pauladg@usal.es (P.D.); mariagg@usal.es (M.G.-G.); 2 Proteomics Unit, Cancer Research Centre (IBMCC/CSIC/USAL/IBSAL), Salamanca 37007, Spain; E-Mails: romade@usal.es (R.M.D.); nibarrola@usal.es (N.I.); 3 Rheumatology Division, ProteoRed/ISCIII Proteomics Group, INIBIC, Hospital Universitario de A Coruña, A Coruña 15006, Spain; E-Mail: llourido@udc.es; 4 Biotechnology National Centre, Spanish National Research Council (CSIC), Madrid 28049, Spain; E-Mail: jcasado@cnb.csic.es; 5 Biodesign Institute, Arizona State University, 1001 South McAllister Avenue, Tempe, AZ 85287, USA; E-Mail: Joshua.Labaer@asu.edu

**Keywords:** protein microarray, NAPPA, high-throughput screening, biomarker, protein-protein interaction, microarray generation

## Abstract

Nucleic Acid Programmable Protein Arrays (NAPPA) have emerged as a powerful and innovative technology for the screening of biomarkers and the study of protein-protein interactions, among others possible applications. The principal advantages are the high specificity and sensitivity that this platform offers. Moreover, compared to conventional protein microarrays, NAPPA technology avoids the necessity of protein purification, which is expensive and time-consuming, by substituting expression *in situ* with an *in vitro* transcription/translation kit. In summary, NAPPA arrays have been broadly employed in different studies improving knowledge about diseases and responses to treatments. Here, we review the principal advances and applications performed using this platform during the last years.

## 1. Introduction

The complexity of the human proteome requires high-throughput (HT) approaches to define its study. During the last decade, protein microarrays have emerged as a useful tool for the analysis of the proteome at large scale [1]. Currently, protein microarrays have been successfully applied in the study of biomarkers, post-translational modifications (PTMs) of proteins, and various types of interactions with proteins. In addition, they have shed light on the biological roles of proteins related to and involved in diseases [2,3,4].

Although recent advances have improved the sensitivity and reproducibility of common and widespread proteomics technologies (such as 2D-GE, MALDI-TOF, or LC-MS/MS), they are not readily implemented in a HT format [5]. In contrast with other proteomic strategies, protein microarrays avoid the need for pre-fractionation of the sample. In fact, complex and non-fractionated proteome mixtures, such as serum, plasma, urine and tissue extracts, can be directly used for experimentation [2]. For this reason, protein microarrays offer a powerful technology for functional proteomics analysis in HT format.

Microarray technologies utilize densely printed micro- or nano-spots of capture ligands immobilized onto a solid support that are exposed to samples containing the corresponding binding molecules (often referred to as queries), allowing the simultaneous analysis of thousands of capture targets within the same assay [6,7]. Thus, protein array technology enables multiplex and highly sensitive protein assays capable of handling and resolving complex proteomes with limited available samples [5,7].

In general, protein microarrays can be prepared in different formats (planar, beads…) and wide diversity of content (from antibodies and recombinant proteins to cell lysates). In fact, researchers have classified protein arrays based on the format or the content; however, in practice, these differences are more related to nomenclature than methods.

The first critical step to build protein microarrays is to display proteins on a solid surface for the detection of their biochemical activities in a multiplex manner. Hence, this is considered one of the challenges in protein microarrays field because of the high variability in biochemical properties (such as oligomerization states, PTMs, stability, affinities and specificities, isoelectric point…).

**Figure 1:**
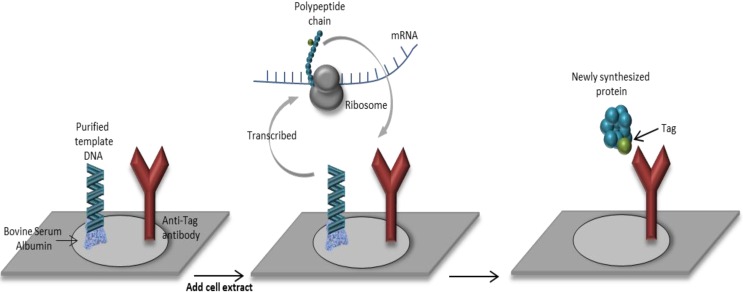
Diagram of Nucleic Acid Programmable Protein Array (NAPPA). Bovine serum albumin is used for printing purified template DNA (including the protein of interest and a tag molecule) onto a slide together with an antibody that recognizes the specific tag. When the cell extract is added, the transcription and translation are initiated and the expressed protein is captured by the anti-tag antibody.

In addition, protein production and purification in a HT manner with high yield can be challenging [8]. This is because cell-based expression systems and the protocols of purification to generate large quantities of proteins are usually very tedious, result in highly variable yields and do not guarantee the protein integrity. These issues represent one of the major bottlenecks in HT functional proteomics studies. In Nucleic Acids Programmable Protein Arrays (NAPPA), the proteins are synthetized from a DNA template directly onto the surface of the array and the nascent protein is captured at the same time by an affinity reagent [9] avoiding the vast majority of drawbacks mentioned above (Figure 1).

Here, we briefly review NAPPA technology and its recent applications in the study of pathologies, discovery of biomarkers and also vaccine development.

## 2. Concept of Protein Microarrays

In 2004, LaBaer’s lab designed and developed a novel protein microarray, termed NAPPA, based on shuttling cDNA clones into expression plasmids—typically using Gateway technology—adding a transcriptional promoter and also an in-frame polypeptide capture tag.

Cloning the cDNAs into a specialized vector requires a much greater upfront investment compared to the conventional Polymerase Chain Reaction (PCR). However, there are several advantages over typical protein microarrays that establish NAPPA technology as a powerful platform: (i) the production of a glycerol stock for the clone allows the maintenance of the gene integrity indefinitely; (ii) it ensures high fidelity since the clone sequence is verified; and (iii) inserting the clones into plasmids permits the incorporation of tags and antibiotic resistance genes for specific selection. Generally, proteins are fused with glutathione-S-transferase (GST) in NAPPA technology; however, other tags such as flag, hemagglutinin (HA), c-Myc, and Halo tags have been used for specific applications.

**Figure 2:**
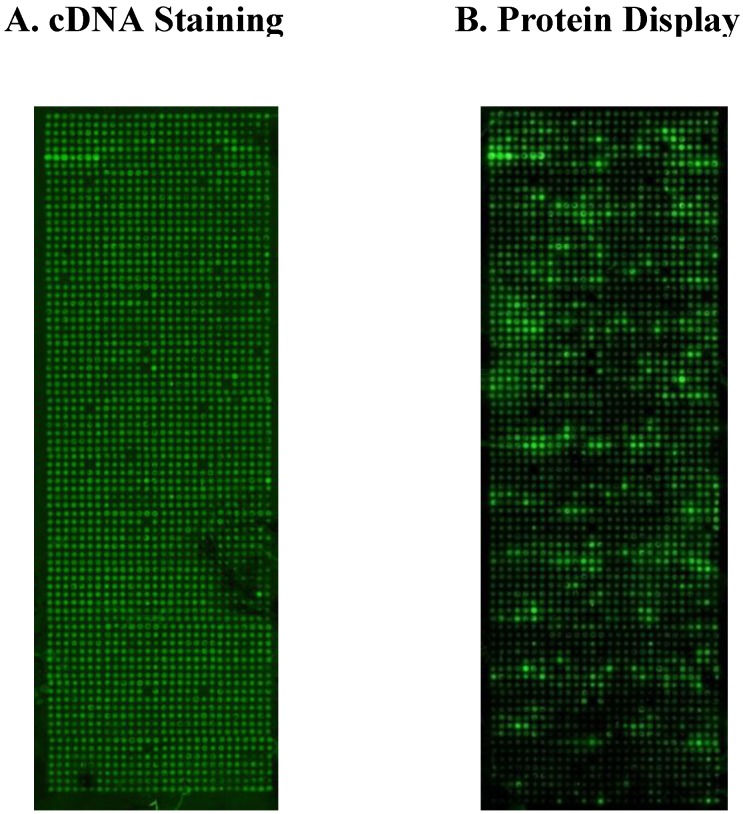
Scanning images showing the spots corresponding to DNA printed onto the surface before the protein expression (**A**) and the spots for the expressed proteins after the incubation with the anti-tag antibody (**B**).

Bacteria cultures are employed as hosts for the high quality supercoiled plasmid DNA of interest. Thus, after the purification of the DNA plasmids, these are printed onto an activated ester surface along with a homo-bifunctional crosslinker (BS^3^, SMCC…), bovine serum albumin (BSA) and anti-tag antibody. BSA efficiently increases the DNA binding and reduces the unspecific interactions, whereas the anti-tag antibody attaches the expressed protein [10]. When the cell-free expression system is added to the array, a coupled transcription/translation reaction results and the nascent protein is linked to the capture agent tag through the C-terminal end assuring the complete translation of the protein (Figure 2). A cell-free expression system produces a protein by using biomolecular translation machinery without the usage of living cells. The reaction solution includes the transcriptional and translational molecular machinery consisting of RNA polymerases for mRNA transcription, ribosomes for polypeptide translation, tRNA and amino acids, enzymatic cofactors, an energy source, and cellular components essential for proper protein folding.

In an updated version of NAPPA, LaBaer and colleagues built an array of 1000 human genes and demonstrated that the vast majority of these genes (~96%) showed a detectable protein signal. In addition, they concluded that this platform is unbiased in relation to protein size—signal intensities were independent from molecular size—enabling unbiased study of protein function in a HT manner. In turn, they demonstrated their high stability since the DNA is more stable than proteins. Moreover, there is high intra- and inter-protein display reproducibility in these kinds of arrays [9]. It is also remarkable that NAPPA is the only *in situ* protein technology that has been widely employed in biological and biomedical research studies. To date, more than 30,000 different proteins have been produced on NAPPA arrays, including whole proteomes of several microorganisms and >12,000 different full-length human proteins. All in all, thousands of NAPPAs can be produced per year thanks to the automation developed in the field.

## 3. Applications of NAPPA Technology

**Figure 3:**
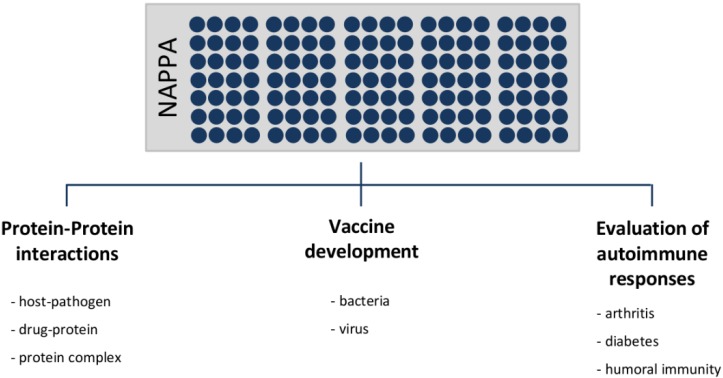
Applications of NAPPA technology.

Next, different applications of NAPPA technology are described showing several studies and their results. These applications are classified according to protein-protein interactions studies, vaccine development and the evaluation of autoimmune responses (Figure 3). Table 1 summarizes the main studies developed in the field and described in the text.

### 3.1. NAPPA Technology for Understanding Proteins-Proteins Interactions

Four years after the first design of NAPPA technology, LaBaer’s group confirmed that protein function is maintained in printed proteins on high-density arrays. With this purpose, they designed an array expressing 647 unique genes in duplicate and tested for several well-characterized interactions, Jun-Fos and p53-MDM2 among others. Simultaneously, they expressed the corresponding protein printed on the array and co-expressed the query protein by adding the appropriate cDNA to the cell-free expression lysate. Using specific antibodies against Jun, Fos and MDM2, they detected specific interactions of these proteins. It is also necessary to take into account that protein function can be compromised by lack of PTMs and/or misfolding of certain domains due to the absence of chaperones and cofactors. Concerning the lack of PTMs, it is possible to use alternative cell free expression systems depending on the protein to be expressed. Thus, different expression systems have been developed (including HeLa, *Leishmania*, *E. coli*, rabbit expression systems, among others). Also, including ribosomal machinery and chaperones (such as HSP90 or HSC70) may encourage the folding of large multi-domain proteins [9].

More recently, in 2012, Fuentes and collaborators published a work in which a total of 450 mRNAs from *O. moubata* tick salivary glands were extracted and purified, and then transfected into a donor vector (pDONR222) generating a library of cDNA. Finally, this library was transfected again into a library destination expression vector (pANT7_GST), which allows *in situ* expression of GST-tagged proteins in cell-free systems. They built a NAPPA array randomly choosing 480 clones with validated sequences. After confirming successful display of the recombinant fused GST tag protein, the correct display of individual tick proteins was also checked with serum recognizing Om44, a P-selectin salivary protein from *O. moubata* whose neutralization induces antibody block tick feeding. To test the functionality of the proteins in the array, they performed protein-protein interaction studies with the recombinant P-selectin/Fc chimera. With this aim, the proteins on the array and P-selectin/Fc chimera were expressed *in situ* normally and also in the presence of canine pancreatic microsome membranes (CMMs). They found that P-selectin/Fc chimera interacted with phospholipase A2 (PLA2) expressed *in situ* on the array. This finding suggested that this secreted *O. moubata* PLA2 (sPLA2) could be a potential P-selectin interacting partner [11].

As another example, a NAPPA array was designed for systematic characterization of viral protein-host interactions. Through the access to viral ORFs in flexible cloning formats, the LaBaer’s lab is releasing the initiation of a panviral proteome collection of 2035 ORF clones from 830 viral genes in the Gateway® recombinational cloning system. In this work, NAPPA arrays are suitable, highly efficient and flexible platforms for displaying viral proteins and detecting host serological responses using micro-fluidic multiplexed immunoassays and allowing the study of host-viral protein interactions [12]. Related to host-pathogen interactions in *Legionella pneumophila* infections, this group have applied NAPPA technology to determine the interaction network of the pathogen with 10,000 unique human proteins. They identified known and novel interaction candidates and, additionally, substrates for an effector with and adenylyl transferase domain that catalyzes AMPylation. Their results highlighted the amenability of NAPPA to high-throughput analysis of effectors from a wide variety of human pathogens [13].

Nicolini and collaborators clinically screened neuro-oncological patients respondent to temozolomide (TMZ) from those showing resistance to the drug by using a NAPPA-based nanoconductometric sensor [14]. Their results shower a properly discrimination of protein-protein interactions depending on the behavior against TMZ [15]. Finally, Liang *et al*. have successfully coupled two different technologies (label-free and real-time detection method plasmonic-based electrochemical impedance microscopy with NAPPA arrays) to determine small molecule binding kinetics. This approach allowed the measurement of binding kinetics and affinity parameters between small molecule drugs (imatinib and SB201290) and their target proteins (kinases ABl1 and p38-α) with high sensitivity and reproducibility. These results demonstrate that NAPPA methodology is a reliable technology to understand small molecules interactions in biological systems and is also useful in the discovery of small molecules drugs [4].

### 3.2. Vaccine Development by NAPPA Technology

Since the development of NAPPA arrays, many research groups have used different NAPPA-based platforms for investigating immune diseases and improving vaccine development. Next, we briefly describe some of these studies.

Respiratory tract and lung infections in cystic fibrosis (CF) patients and individuals who are otherwise immune compromised can be caused by a gram-negative bacterium called *Pseudomonas aeruginosa*. This microorganism was selected by Montor *et al*. [16] to test candidate membrane antigens with NAPPA arrays. The principal goal of their work was to map the immune responses of patients infected with *P. aeruginosa* to determine which bacterial outer membrane proteins induced a strong immune response. The principal difficulty in purifying membrane proteins to display on NAPPA arrays is related to their hydrophobic domains. They designed a NAPPA array containing all 262 outer membrane proteins of the bacterium. Serum samples from 22 CF patients with documented pseudomonal pneumonia and 16 non-CF individuals with various acute *P. aeruginosa* infections as well as 15 healthy controls were selected for array screening. After analysis, 12 proteins triggering an adaptive immune response were identified in a majority of the infected patients, yielding valuable information about which bacterial proteins are recognized by the immune system during the natural course of infection.

In turn, Ceroni and colleagues [17] used NAPPA arrays for a systematic analysis of the IgG antibody immune response against varicella zoster virus (VZV), a human herpes virus, encoding at least 69 distinct viral proteins, which causes chickenpox after primary infection and shingles during reactivation. Its effects are particularly important in pregnancy and immunocompromised patients and sera-diagnostic tests are commonly used for its detection. In order to investigate the humoral immune response to VZV infection or vaccination in more detail, Ceroni developed a specific NAPPA array containing all 69 VZV proteins mentioned above and performed a detailed analysis of 68 sera from individuals with either no infection or an acute VZV infection. The obtained results confirmed previous knowledge about viral open reading frames (ORF) such as reactive glycoproteins antigens (ORF 5, ORF 14, ORF 31, ORF 37, ORF 68), and also found novel responses against a variety of other membrane proteins (ORF2, ORF24), capsid (ORF20, ORF23, ORF43) and tegument (ORF53, ORF9, ORF11), as well as others related to virus replication (*i.e*., ORF 25, ORF26, ORF28) and transactivator proteins (ORF12, ORF62 and ORF63).

### 3.3. Evaluation of Autoimmune Responses

In autoimmune diseases, antibodies, known as “auto-antibodies,” are often generated by the humoral immune system against self-proteins in response to many pathological processes. This kind of antibody follows a specific pathway to recognition by the immune system, including antigen over-expression, mutation, and/or altered PTM released from damaged tissues [18,19]. The presence of these autoantibodies is related to the development of certain diseases such as diabetes. Thus, they can be useful as diagnostic/prognostic biomarkers [20]. As diagnostics they have several key advantages: (i) they can be detected even before the appearance of clinical symptoms; (ii) even if the antigen that induced them is absent or present at very low amounts, the antibodies can be readily detected; (iii) they can be measured from easily obtained sources such as blood other body fluids; (iv) they are very stable in standardized collection vessels; and (v) they are straightforward to measure using many available chemistries.

In 2007 Anderson and colleagues employed NAPPA arrays for serological screening in breast cancer. After NAPPA design, they tested p53 together with other three negative control antigens (S100A7, p21 and ML-IAP) with positive and negative p53 sera confirming the expression for all the proteins printed and checking the detection of antibodies against p53. Moreover, they determined differences in p53-expression levels between healthy donors and breast cancer patients, and also within disease stages. In addition, they confirmed that many regions of the protein expressed were accessible on the arrays. To extend the study to autoantibody biomarker detection, they built a high density NAPPA array printing 1117 cancer related genes of which 539 were implicated on breast cancer and tested them against melanoma, ovarian and breast cancer sera [21]. Later, they increased the number of novel autoantibodies to be tested in breast cancer (around 4988 candidate antigens). Finally they identify 28 autoantibodies that could distinguish between benign breast disease and invasive cancer in a blinded study [22].

Recently, LaBaer *et al*. developed several NAPPA studies for juvenile idiopathic arthritis (JIA) [23] and type 1 diabetes (T1D). In both cases, serological autoantibodies (AAbs) from the disease were screened using a two-stage method. Firstly, more than 6000 unique proteins were displayed in NAPPA arrays which were incubated with 50 sera from T1D patients and 20 from controls allowed the elimination of uninformative antigens. In the second stage, 750 remaining genes were printed in duplicate and 26 proteins were identified as novel AAbs (TBCA, CDK4, CDK6, TBRG4, among others) with *p* < 0.005 [24]. For juvenile idiopathic arthritis, they assessed the levels of antibodies present in the systemic circulation and synovial joint of a small cohort of juvenile arthritis patients as a pilot study. Their results showed a strong correlation between the circulating antibody levels and those of the inflamed joint.

In 2009, Wong *et al*. adapted NAPPA technology to the Luminex suspension bead array platform to monitor the humoral immunity. To accomplish this, they expressed the proteins and captured them with the Luminex beads through anti-tag antibodies. After mixing the antigen-loaded beads, serum was added and human IgG was detected with standard secondary detection reagents. Protein arrays are a useful method for testing a moderate number of clinical samples against thousands of candidate proteins. The advantage of the Luminex approach is that it allows testing hundreds of clinical samples against a moderate number of candidate antigens, *i.e*., mesoscale. They concluded that detection of antibodies was highly reproducible and the specificity and limits of detection of the platform were comparable to standard ELISAs [25].

Recently, Henjes and Lourido have performed an analysis of auto-antibody profiles in osteoarthritis (OA) using comprehensive protein arrays concepts. In this work, NAPPA arrays and antigen arrays have been used to characterize differential autoantibody profiles in a set of 62 samples from OA, rheumatoid arthritis (RA), and healthy controls. An untargeted screen was performed on 3840 protein fragments spotted on planar antigen arrays, and 373 antigens were selected for validation on bead-based arrays. In the NAPPA approach, a targeted screening was performed on 80 preselected proteins. The autoantibody targeting CHST14 was validated by conventional ELISA assays in the same set of patients. Altogether, nine and seven disease-related autoantibody target candidates were identified, respectively, and this work demonstrated a combination of these two array concepts for biomarker discovery and their usefulness for characterizing disease-specific autoantibody profiles [26].

## 4. Recent Technical Advances to the Platform

Several developments in protein array technology have allowed an improvement in throughput and sensitivity, achieving better capture and probing of proteins.

For instance, Wang and colleagues have described the use of human cell-free lysates (HeLa cell lysate as an *in vitro* transcription/translation system) in NAPPA arrays for protein expression enhancing protein yield and for presenting both natural and denatured forms of proteins for antibody biomarker discovery. Through their results, they demonstrated that autoantibody profiles from denatured protein arrays were distinct from those native protein arrays when probing with plasma samples. Furthermore, they blocked the protein arrays with *E. coli* lysates, reducing the background and improving the antibody signals [27]. Another study from the same lab reported an improvement in protein display by using the human cell-free lysate (10-fold higher) compared to the conventional rabbit reticulocyte lysate [28]. In turn, Xiabo and collaborators used NAPPA technology for the detection of global pathogen-host AMPylation (adenylytation PTM). Specifically, they developed a novel nonradioactive AMPylation screening platform using high-density cell-free protein microarrays for the screening of 10,000 unique human proteins with *Vibrio parahaemolyticus* and *Histophilus somni*, identifying new AMPylation substrates (including Rac 2 and Rac 3) [29].

Collaboration between the Nicolini and LaBaer groups reported an innovative kind of NAPPA platform, in which the cDNA includes the SNAP tag and the expression is performed by using the PURE system (reconstituted from the purified components necessary for *E. coli* translation). Their principal purpose was to achieve the combination of mass spectrometry and fluorescence technology for protein microarrays. Their results with the PURE system showed a protein yield about 20 times higher with respect to the rabbit reticulocyte expression system [30].

Additionally, LaBaer’s lab has developed a very high density NAPPA array without any diffusion or contamination between spots by depositing the samples on 8000 nano-volume wells and, recently, the protein expression lysate into each well to achieve the protein expression in a HT manner. Furthermore, they present preliminary results with an ultra-high density protein array including up to 24,000 nanowells [31].

In turn, Nicolini and collaborators demonstrated the effective use of label-free approaches (anodic porous alumina, APA; and atomic force microscopy, AFM) in combination with NAPPA technology to test the expression and the atomic structure of proteins of interest. These studies show the possibility of overcoming limitations at the fluorescence detection level [32].

**Table 1:** Summary of NAPPA protein microarray applications.

Description	Aim/Results	Reference
High density array (1000 human genes/array)	96% detectable signa lUnbiased to protein size No difference or contamination between spots	[9,31]
450 mRNA *O. moubata* tick salivary glands	P-selectin/Fc chimera interaction with phospholipase A2	[11]
Systematic characterization of viral protein-host interactions	Panviral Proteome Collection	[12]
Neuro-oncological patients respondent to TMZ	Discrimination of protein-protein interactions	[14,15]
Label-free techniques coupled to NAPPA	Determination of small molecule binding proteins	[4]
Respiratory tract and lung infections in cystic fibrosis	To test candidate membrane antigens	[16]
Analysis of IgG antibody immune response against VZV	To identify known and novel antigens	[17]
Serological screening in breast cancer	Protein profiling to distinguish benign breast disease and invasive cancer	[21]
Juvenile idiopathic disease and type 1 diabetes	To screen for disease-specific autoantibodies in plasma samples.	[23]
NAPPA coupled to Luminex suspension bead array platform	To monitor the humoral immunity	[25]
Osteoarthritis and rheumatoid arthritis	To characterize differential autoantibody profiles	[26]
Use of HeLa cell-free lysates	To enhance protein yield	[27]
A nonradioactive AMPylation screening platform using high-density cell-free protein microarrays	To identify novel substrates of AMPylators with different domains or in different species	[29]
SNAP tag	Combination of MS/MS and fluorescence technology	[30]
APA and AFM coupled to NAPPA	To test the expression and atomic structure of proteins	[32]

*TMZ*, temozolomide; *NAPPA*, Nucleic Acid Programmable Protein Array; *VZV*, varicella zoster virus; *APA*, anodic porous alumina; *AFM*, atomic force microscopy.

## 5. NAPPA Alternative Methods

Besides NAPPA technology, several *in situ* expressed microarrays have been developed, such as protein *in situ* arrays (PISA) and printing protein arrays from DNA (DAPA). The main difference between PISA and NAPPA is that the DNA template is added as a free molecule together with the reaction mixture. Thus, it is not necessary to immobilize the DNA onto the surface [33]. Angenendt and colleagues demonstrated that yield signals for protein expressed with these arrays were comparable to 300 μg/mL directly spotted proteins. Moreover, the volume of required sample was too low (subnanotliter volumes) and the nature of the surfaces determined the protein binding. Thus, the nickel chelate-coated slides generated an unspecific binding. Finally, they refined and miniaturized PISA arrays by using multiple spotting technique to get a high-density protein microarray with up to 13,000 spots [34].

On the other hand, the DAPA strategy, developed in 2007, is characterized by assembly face-to-face between a slide containing the DNA templates for the proteins and a second slide pre-coated with a protein-capturing reagent. For the transcription/translation, the cell-free system is placed between the two slides using a cell-extract soaked membrane [35,36]. Although this is the basis of the technology, modifications can be made to improve its functionality. In such a way, Schmidt and colleagues [37] have investigated the influence of different support coatings (Ni-NTA, Epoxy, 3D-Epoxy and Polyethylene glycol methacrylate (PEGMA)) concluding that their optimal combination results in high protein yields and optimized spot morphology. Moreover, using a tag-specific capture antibody on a protein repellent surface coating, they improved the specificity of protein capturing and obtained amounts of expressed proteins comparable to classical protein arrays.

Finally, ProtoArrays are also a remarkable microarray approach. They are characterized for including thousands of proteins (>9000) in a high-density array allowing a high-throughput screening using low volumes of sample (~10 μL of serum). Several studies have been performed by using these arrays. For instance, they are employed for autoimmune antibody screening studies to discover biomarkers of the Parkinson’s disease (ParkCHIP). In this research, Turewicz and collaborators [38] studied a large cohort of samples and adapted the default workflow for these arrays to their requirements. This constitutes a clear example of the flexibility of this approach. Furthermore, these ProtoArrays have been employed to study systemic erythematosus lupus (SLE), specifically to identify novel autoantibodies associated with the disease. In total, 9500 antigens were screened resulting in 446 IgG and 1218 IgM autoantibodies significantly elevated in SLE patients. In this research, the researchers not only identified previously described autoantibodies (SSA/SSB, Sm/RNP…) but also reported novel antigens associated with the nucleus, cytoplasma or membrane [39].

## 6. Conclusions and Future Perspectives

The development of new strategies for protein profiling may improve analysis and reduce the time for screening of thousands of proteins simultaneously. In addition, the expression of the proteins at the moment of usage improves the quality of the proteins, since the final product does not suffer any change related to temperature, pH or degradation. The use of mammalian ribosomes and chaperone proteins contributes to accurate protein folding. Unlike conventional protein arrays, nucleic acid programmable protein arrays (NAPPA) are DNA-based arrays that convert into protein microarrays. A key advantage of this approach is its adaptability. To display a different set of proteins—the proteins of a recently discovered pathogen, a set of proteins related to a gene family, or a series of mutant versions of a protein of interest—the practitioner merely has to produce the DNA clones encoding the proteins. There is no need to go through a long development process of purifying the proteins. To produce the protein of interest, the coding-DNA inserted into a plasmid is printed onto a surface and then is transcribed/translated by using a cell-free expression system. Moreover, once printed, the coding material remains intact, even at ambient temperature, thanks to the high stability of nucleic acids. The labile protein is not expressed until it is required.

Many investigators have employed this NAPPA approach for their studies, including cancer, autoimmune diseases, host-pathogens interactions, quantification of protein binding kinetics, and infection responses to microorganisms showing a high specificity and selectivity with accurate and reproducible results. Moreover, it has been demonstrated that the platform is unbiased and independent of molecular size or protein family type.

In summary, NAPPA technology seems to be a powerful tool for performing HT analysis in different formats (planar, beads…), with different samples (sera, urine, saliva…) and combined with other platforms. All in all, clinical and diagnostic screenings may be accomplished in a rapid and reliable manner.
